# CASPER: context-aware scheme for paired-end reads from high-throughput amplicon sequencing

**DOI:** 10.1186/1471-2105-15-S9-S10

**Published:** 2014-09-10

**Authors:** Sunyoung Kwon, Byunghan Lee, Sungroh Yoon

**Affiliations:** 1 Bioinformatics Institute, Interdisciplinary Program in Bioinformatics, Seoul National University, 1 Gwanak-ro, Gwanak-gu, 151-747 Seoul, Korea; 2 Department of Electrical and Computer Engineering, Seoul National University, 1 Gwanak-ro, Gwanak-gu, 151-744 Seoul, Korea

**Keywords:** paired-end merging, amplicon sequencing, high-throughput short-read sequencing, sequence analysis

## Abstract

Merging the forward and reverse reads from paired-end sequencing is a critical task that can significantly improve the performance of downstream tasks, such as genome assembly and mapping, by providing them with virtually elongated reads. However, due to the inherent limitations of most paired-end sequencers, the chance of observing erroneous bases grows rapidly as the end of a read is approached, which becomes a critical hurdle for accurately merging paired-end reads. Although there exist several sophisticated approaches to this problem, their performance in terms of quality of merging often remains unsatisfactory. To address this issue, here we present a **c**ontext-**a**ware scheme for **p**aired-**e**nd **r**eads (CASPER): a computational method to rapidly and robustly merge overlapping paired-end reads. Being particularly well suited to amplicon sequencing applications, CASPER is thoroughly tested with both simulated and real high-throughput amplicon sequencing data. According to our experimental results, CASPER significantly outperforms existing state-of-the art paired-end merging tools in terms of accuracy and robustness. CASPER also exploits the parallelism in the task of paired-end merging and effectively speeds up by multithreading. CASPER is freely available for academic use at http://best.snu.ac.kr/casper.

## Introduction

The advent and widespread use of next-generation sequencing (NGS) [1-3] has posed new challenges and opportunities for informatics [4,5] due to the high-throughput nature and the relatively short and noisy reads compared to the traditional Sanger sequencing. NGS thus sparked the development of new pipelines (for base-calling, genome assembly/mapping, and other essential tasks) that consider the characteristics of the NGS platforms used.

In paired-end sequencing, a DNA fragment is read from either end of the fragment, and some NGS platforms (such as Illumina HiSeq, MiSeq, and GAIIx) inherently generate paired-end reads [2,3]. When a fragment is larger than the sum of the forward and reverse reads, there exists a gap between the two reads [6]. Otherwise, the forward and reverse reads overlap, which can ideally give the effect of elongating reads. Having longer reads provides many benefits to downstream tasks in the informatics pipeline [7,6,8]. The read length of today's sequencers tends to continuously increase: *e.g*., at the time of writing, Illumina MiSeq can produce 2 × 300 bp reads using its reagent kit v3 (http://www.illumina.com).

**Figure 1:**
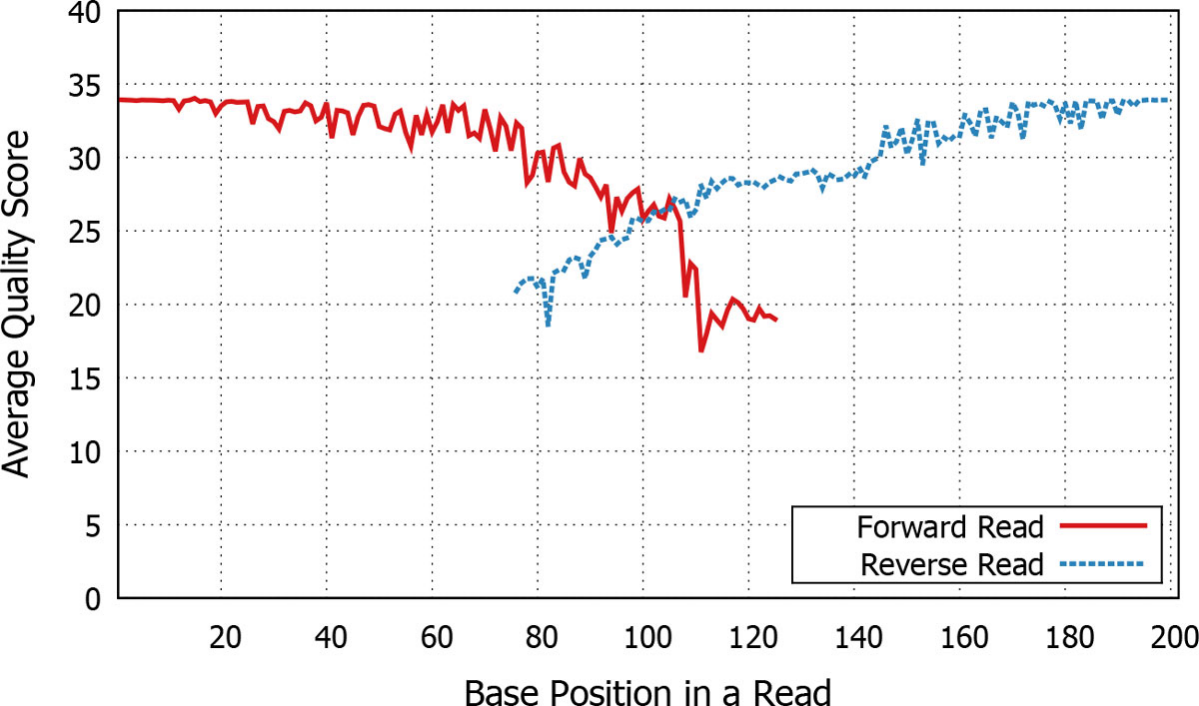
**Overlap between paired-end reads**. Next-generation sequencing techniques including the Illumina platform tend to result in rapid degradation of the sequencing quality as the end of a read is approached [12]. Consequently, the overlapping region (formed by the ends of forward and reverse reads) in a paired-end read frequently contains errors originating from sequencing and/or base-calling.

However, NGS techniques including the Illumina platform tend to result in rapid degradation of the sequencing quality as the end of a read is approached (Figure 1). As a result, the overlapping region (formed by the ends of forward and reverse reads) in a paired-end read frequently contains errors originating from sequencing and/or base-calling. Sequencing results are often annotated with per-base *quality scores *representing the error probability [9].

Such limitations of sequencing technology often prevent the accurate merging of paired-end reads, so the pursuit of new tools for the reliable merging of overlapping paired-end reads has become an active area of research, *e.g*., SHERA [7], FLASH [6], PANDAseq [10], and COPE [11]. Most of these tools start by finding the best overlap between a pair of forward and reverse reads and then try to merge them by resolving mismatching bases in the overlap. The best overlap is sought by considering the overlap alignment [7], the fraction of (mis)matching bases [6,11], or quality scores [10,11]. The mismatch resolution is mostly achieved by considering quality scores [6,10] and replacing the base with the lower quality score by the base with the higher quality score. Quality-score-based resolution often produces incorrect results, especially when the quality scores of mismatching bases do not differ significantly [12]. It was proposed to use quality scores and *k*-mer frequency together as the merge criterion [11], but the resulting methodology tends to be time-consuming and of unsatisfactory accuracy according to our experience. Due to the importance of paired-end merging, some sequence assemblers (*e.g*., ALLPATHS-LG [13]) contain a module for merging paired-end reads as a preprocessing stage, although the performance, flexibility, and applicability of such internal modules tends to be limited compared to the aforementioned methods dedicated to paired-end merging.

To overcome the limitations of the current approaches to merging paired-end reads from amplicon sequencing, here we propose a computational method called *context-aware scheme for paired-end reads *(CASPER). In this scheme, when the difference between the quality scores of mismatching bases is significant, CASPER relies on the quality scores for correction. If not, CASPER instead examines *k*-mer-based contexts around the mismatch and makes a statistical decision (up to *k *partial decisions for each mismatch). CASPER then makes a final decision based on the ensemble of the earlier partial decisions. According to our experiments, CASPER significantly outperforms the existing approaches in terms of accuracy of merging and resilience to noise. Furthermore, the time demand of CASPER remains reasonable in most cases, taking only a few tens of seconds to process one million reads. CASPER is freely available for academic use at http://best.snu.ac.kr/casper.

## Proposed method

**Figure 2:**
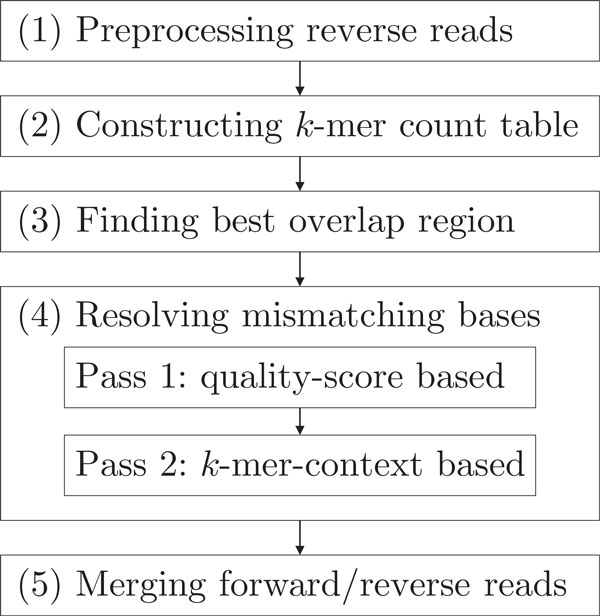
**Overall flow of CASPER**. The proposed CASPER methodology consists of five main steps: (1) preprocessing, (2) constructing a table of *k*-mer counts, (3) finding the best overlap position, (4) resolving mismatches in the overlap, and (5) merging forward and reverse reads.

Figure 2 shows the overall flow of the proposed CASPER approach that consists of five main steps: (1) preprocessing, (2) constructing a table of *k*-mer counts, (3) finding the best overlap position, (4) resolving mismatches in the overlap, and (5) merging forward and reverse reads. Before explaining the details of each step, we present the assumptions and definitions to be used in the explanation.

In paired-end sequencing, the sequencer produces two reads for each DNA fragment. Suppose that the fragment length is *m *(bases), and the lengths of forward and reverse reads are both *n*. An overlap between the two reads occurs when *m <*2*n*. The sequence of a fragment is denoted by *S *= 〈*s*_1_*, s*_2_*, . . . , s_m_*〉, *s_i _*ϵ {A, C, G, T}. We denote the forward and reverse reads from *S *by *X *= 〈*x*_1_*, x*_2_*, . . . , x_n_*〉 and *W *= 〈*w*_1_*, w*_2_*, . . . , w_n_*〉, respectively. The *i*-th base in *X *(*i.e*., *x_i_*) is also denoted by *X*(*i*), and the subsequence of *X *ranging from the *i*-th to *j*-th bases (*i.e*., 〈*x_i_, . . . , x_j_*〉) is denoted by *X*(*i *: *j*).

With no sequencing error (*e.g*., substitution, insertion, and deletion), *x_i _*= *s_i _*and ${\bar{w}}_{i}=s_{m-i+1}$ for 1 *≤ i ≤ n*, where ${\bar{w}}_{i}$ represents the Watson-Crick complementary base of ${\bar{w}}_{i}$ (*e.g*., Ā is T, *vice versa*). In practice, bases may be called incorrectly, and each called base is accompanied by a (Phred) *quality score *denoted by *Q *and defined as *Q *= *−*10 log_10_*p*, where *p *is the probability that the corresponding base call is incorrect [9]. Sequences *X *and *W *are assumed to be accompanied by $Q_{X}=\left\langle q_{x_{1}},q_{x_{2}},\ldots ,q_{x_{n}}\right\rangle$ and $Q_{W}=\left\langle q_{w_{1}},q_{w_{2}},\ldots ,q_{w_{n}}\right\rangle$, respectively. Symbol 'N' represents any base (indecisive base-call) and is normally accompanied by the lowest quality score available. We assume that there are few indel-type sequencing errors, as is commonly the case with the Illumina platform.

### Preprocessing, *k*-mer counting, and overlap detection (steps 1-3)

In the first step of CASPER, every reverse read *W *in the input is preprocessed to facilitate the downstream steps. Specifically, CASPER reverses the order of bases in *W *and then complements each base. *W *and *Q_W _*are converted to *Y *= 〈*y*_1_*, y*_2_*, . . . , y_n_*〉 and $Q_{Y}=\left\langle q_{y_{1}},q_{y_{2}},\ldots ,q_{y_{n}}\right\rangle$, respectively. $y_{i}={\bar{w}}_{n-i+1}$ and $q_{y_{1}}=q_{w_{n-i+1}}$ for 1 *≤ i ≤ n*. In the remainder of this paper, the term 'reverse read' refers to *Y *instead of *W*.

In the second step of CASPER, a table of *k*-mer counts is constructed from the input reads, where *k *is a user-specified parameter (Table 1). There has been active research on efficient *k*-mer counting. For instance, Jellyfish [14] provides a time-efficient, parallel solution to *k*-mer counting. Methods focused on memory efficiency also exist, *e.g*., bloom-filter-based BFcounter [15] and DSK [16]. In CASPER, we adopt and customize Jellyfish for building a table of *k*-mer counts. We denote this table by *T_k _*. For *k*-mer *X*(*i *: *i *+ *k − *1) = (*x*_*i*_, *x*_*i*+1_, . . . , *x*_*i*+*k−*1_), *T_k _*[*X*(*i *: *i *+ *k − *1)] indicates the number of occurrences of this *k*-mer in all forward and reverse reads of the input data.

**Table 1:** User-specified parameters of CASPER and the default values used for experiments

Parameter	Default	Description
*K*	17	The size of *k*-mers (in bp) used to represent contexts around mismatching bases
*Ω*	10	The minimum length (in bp) of the overlap between forward and reverse reads
*γ*	0.5	CASPER abandons merging if the mismatch ratio in the overlap is greater than *γ*
*δ*	19	Context-based mismatch resolution starts if quality scores differ less than *δ*

Algorithm 1 shows pseudo-code of the remaining steps of CASPER. In the third step of CASPER, it is decided as to how much it needs to shift (to the right) the reverse read *Y *with respect to the forward read *X *in such a way that the fraction of mismatching bases in the overlap region is minimized (lines 1-8). The resulting overlap region is considered the best. The (mis)match ratio is widely used in the literature to locate the optimal overlap between paired-end reads [6,11]. Parameter *ω *specifies the minimum length of an overlap. If too many mismatches exist in the overlap (*i.e*., the mismatch ratio exceeds a user-specified 'give-up' threshold *γ*), CASPER does not merge the reads (line 9).

### Resolving mismatching bases in forward and reverse reads (step 4)

In the fourth step of CASPER, the mismatching bases in the overlap region are corrected. Ideally, for each position of the overlap region, the base in the forward read should match the base in the reverse read. Due to experimental error and other non-idealities, however, these two bases often mismatch. To merge paired-end reads successfully, we need to resolve mismatching bases. The basic principle is simple: overwrite the incorrect base with the correct base, assuming that the base either in forward or reverse read is correct. The remaining question is which of the two bases is correct. Lines 10-28 in Algorithm 1 reveal how CASPER answers this question. CASPER scans the overlap region twice, once for quality-based resolution and once more for context-based resolution.

Note that in lines 11-28 of the code, position *i *in forward read *X *corresponds to position *i′ *in reverse read *Y *. We assume that the two bases *X*(*i*) and *Y *(*i′*) are not 'N' (if both *X*(*i*) and *Y *(*i′*) are 'N', then they are skipped; if either one is 'N', then the other base is informative).

#### First pass: quality-score-based correction (lines 10-14)

For each pair of mismatching bases in the overlap between the forward and reverse reads, CASPER first considers the difference in their quality scores. For *X*(*i*) and *Y *(*i′*), let *b_h _*(*b_l_*) denote the base with the higher (lower) quality score. For a user-specified parameter *δ*, if *|Q_X _*(*i*) *− Q_Y _*(*i′*)*| > δ*, then CASPER replaces *b_l _*by *b_h_*. The rationale behind this is that a significantly higher quality score of *b_h _*is a strong indicator of its correctness. This is in fact the basis on which most of the current approaches to paired-end merging are grounded. CASPER is differentiated from them by the second step.

#### Second pass: context-based correction (lines 15-28)

A more interesting scenario arises when the difference in quality scores is moderate. In such cases, CASPER no longer makes decisions based on the quality scores, since we cannot assume that the error probability of *b_h _*is negligible relative to that of *b_l _*[12]. For the cases where *|Q_X _*(*i*) *− Q_Y _*(*i′*)*| ≤ δ*, CASPER therefore makes context-based decisions by examining the bases before and after the mismatching position, instead of relying on quality scores.

**Figure 3:**
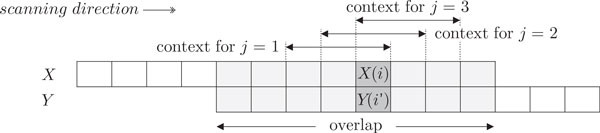
**Definition of *k*-mer context **$C_{j}\left(i,i^{\prime }\right)$. For bases *X*(*i*) and *Y *(*i′*), we consider *k *different (but progressively overlapping) windows along the reads and define the *j*-th context (for 1 *≤ j ≤ k*) in terms of two subsequences of *X *and *Y *. The illustration is for *k *= 3 (1 *≤ j ≤ *3).

For bases *X*(*i*) and *Y *(*i′*), we consider *k *different (but progressively overlapping) windows along the reads and define the *j*-th context (for 1 *≤ j ≤ k*) in terms of two subsequences of *X *and *Y *:

$$C_{j}\left(i,i^{\prime }\right)=\left\langle X\left(i-k+j:i+j-1\right),Y\left(i^{\prime }-k+j:i^{\prime }+j-1\right)\right\rangle$$

as depicted in Figure 3. For each *j*, CASPER estimates $P\left\{X\left(i\right)|C_{j}\left(i,i^{\prime }\right)\right\}$ and $P\left\{Y\left(i^{\prime }\right)|C_{j}\left(i,i^{\prime }\right)\right\}$ and then constructs a *k*-dimensional decision vector *D *= 〈*d*_1_*, d*_2_*, . . . , d_j _, . . . , d_k_*〉, where *d_j _*is defined as

(1) $$d_{j}=\left\{\begin{array}{cc} 1, & \text{if}\;P\left\{X\left(i\right)|C_{j}\left(i,i^{\prime }\right)\right\}>\\ & \;\;\;\;P\left\{Y\left(i^{\prime }\right)|C_{j}\left(i,i^{\prime }\right)\right\};\\ 0, & \text{otherwise}\text{.} \end{array}\right)$$

Element *d_j _*= 1(0) represents that the base in the forward (reverse) read is correct. Note that this decision rule is based on the Bayesian decision theory [17], and thus the error involved in decision *dj *is given by $\varepsilon_{j}=\text{min}\left(P\left\{X\left(i\right)|C_{j}\left(i,i^{\prime }\right)\right\},P\left\{Y\left(i^{\prime }\right)|C_{j}\left(i,i^{\prime }\right)\right\}\right)$.

In other words, CASPER makes a series of preliminary decisions and stores the results in *D*. The elements in *D *can be considered as the outputs from *k *classifiers. We can make a final decision based on *D *using ensemble learning techniques that can combine results from multiple learners [18]. CASPER employs the idea of *linear opinion pools *to make a final decision:

(2) $$\text{choose}\;\;\left\{\begin{array}{cc} X\left(i\right) & \text{if}\;\frac{1}{k}\sum_{j=1\;\;}^{k}d_{j}>0.5\\ Y\left(i^{\prime }\right) & \text{otherwise} \end{array}\right)$$

which can be implemented as voting. If *d_j _*were i.i.d., then the error from the final decision would be *ε_j_/k *[18], but here the *d_j_*'s have dependence on each other originating from the definition of the context, and we expect a higher level of error than the theoretical estimate (see 'Experiments on the context definition and probability computation' in Results and Discussion).

CASPER estimates the probabilities in Eq. (1) using the *k*-mer-count table *T_k _*constructed in step 2. By Bayes' theorem, we can express these probabilities as

$$P\left\{X\left(i\right)|C_{j}\left(i,i^{\prime }\right)\right\}=\frac{P\left\{C_{j}\left(i,i^{\prime }\right)|X\left(i\right)\;\text{is}\;\text{correct}\right\}P\left\{X\left(i\right)\;\text{is}\;\text{correct}\right\}}{P\left\{C_{j}\left(i,i^{\prime }\right)\right\}}$$

and

$$P\left\{Y\left(i\right)|C_{j}\left(i,i^{\prime }\right)\right\}=\frac{P\left\{C_{j}\left(i,i^{\prime }\right)|Y\left(i^{\prime }\right)\;\text{is}\;\text{correct}\right\}P\left\{Y\left(i^{\prime }\right)\;\text{is}\;\text{correct}\right\}}{P\left\{C_{j}\left(i,i^{\prime }\right)\right\}}.$$

Note that the context-based mismatch resolution step assumes that the bases in each of the forward and reverse reads have a similar probability of being correct. This assumption further gives

(3) $$\frac{P\left\{X\left(i\right)|C_{j}\left(i,i^{\prime }\right)\right\}}{P\left\{Y\left(i^{\prime }\right)|C_{j}\left(i,i^{\prime }\right)\right\}}\approx \frac{P\left\{C_{j}\left(i,i^{\prime }\right)|X\left(i\right)\;\text{is}\;\text{correct}\right\}}{P\left\{C_{j}\left(i,i^{\prime }\right)|Y\left(i^{\prime }\right)\;\text{is}\;\text{correct}\right\}}$$

(4) $$\approx \frac{T_{k}\left[X\left(i-k+j:i+j-1\right)\right]}{T_{k}\left[Y\left(i^{\prime }-k+j:i^{\prime }+j-1\right)\right]}$$

(see Results and Discussion for our experimental result that supports the above formulation).

Thus, we can rewrite the decision rule in Eq. (1) using *k*-mer counts as follows:

(5) $$d_{j}=\left\{\begin{array}{cc} 1, & \text{if}\;T_{k}\left[X\left(i-k+j:i+j-1\right)\right]>\\ & \;\;\;\;T_{k}\left[Y\left(i^{\prime }-k+j:i^{\prime }+j-1\right)\right];\\ 0, & \text{otherwise}\text{.} \end{array}\right)$$

In certain cases, the number of partial decisions made per mismatch can be less than *k*. CASPER skips those context windows whose span exceeds the read boundary (lines 20-21). Additionally, when the rightmost position of a window spans a pair of mismatching bases for *j *= *j*^∗ ^*>*1 (*i.e*., *X*(*i *+ *j − *1) *≠ Y *(*i′ *+ *j − *1)), CASPER skips all of the following contexts for *j ≥ j*^∗ ^(line 22). Some bases appearing in such contexts may be erroneous, since they are yet to be corrected. In contrast, it is guaranteed that *X*(*i − k *+ *j *: *i − *1) = *Y *(*i′ − k *+ *j *: *i′ − *1) for 1 *≤ j < j*^∗^, because the mismatch resolution starts with the leftmost position in the overlap and proceeds to the right.

Lines 18-28 of Algorithm 1 implement the decision rules represented by Eq. (2) and Eq. (5), also considering the cases in which the number of partial decisions is less than *k*.

### Additional details

In the final step of CASPER, each pair of forward and reverse reads is merged to produce virtually elongated reads (lines 29-31). The quality score of a base not processed by the mismatch resolution remains unchanged. For a newly replaced base, the quality score of the replacing base is used.

Assuming sequential execution, the worst-case time complexity of CASPER is *O*(*m *log *m*+*N n*^2^), where *N *is the total number of paired-end reads, *n *is the length of each read, and *m *is the number of *k*-mers stored in the *k*-mer table. The worst-case space complexity is *O*(*m *+ *N n*). Note that the *O*(*m *log *m*) time and *O*(*m*) space complexity terms are due to the Jellyfish algorithm [14] we utilize for *k*-mer counting. By parallelization, the *O*(*N n*^2^) term in the time complexity becomes *O*(*N n*^2^*/t*), where *t *is the number of threads used.

There are two main parts of CASPER that are suitable for parallelization: *k*-mer counting and the two-step merging process. Counting *k*-mers in different reads or merging different pairs of reads provides ample opportunities for data-level parallelization. The former is handled by the parallel implementation of Jellyfish. For the latter, we implement the proposed merging algorithm using OpenMP (http://www.openmp.org), an API that supports shared-memory multi-threaded programming.

One might consider making a context-based decision using two separate *k*-mer tables for forward and reverse reads. The context-based decision rule in Eq. (1) will then use *P *{*X*(*i*)*|X*(*i − k *+ *j *: *i *+ *j − *1)} and *P *{*Y *(*i′*)*|Y *(*i′ −k *+*j *: *i′ *+*j −*1)}, reflecting respectively the forward and reverse contexts. This would make the probability estimation more complicated, since the normalization becomes different. Ideally, the forward and (preprocessed) reverse reads should bear the same information and can be considered two replicates of a fragment. Thus, considering the two reads independently may lose the advantage of having such replicates. According to our experiments, this separate-*k*-mer-table approach, while taking more time than the current implementation of CASPER, has a negligible impact on its performance.

## Results and discussion

We test the proposed CASPER methodology with the seven datasets listed in Table 2. To generate A4, A5, S4, and S5, we customize the GemSIM sequencing simulator [19] and apply it to a public dataset [20] using the Illumina error models v4 and v5. Note that datasets A4 and S4 are generated with higher error rates than A5 and S5. In S4 and S5, there is a single reference fragment, whereas A4 and A5 have all of the twenty three reference sequences used in the original publication. Datasets C1, C2, and PA are from real sequencing experiments on bacterial 16S ribosomal RNAs [21,10].

**Table 2:** Details of the datasets used for experiments

dataset type	ID	target†	# Total reads	# Refs^‡^	Fragment length^§^	Read length	Overlap length	Simulator (error model) or sequencer used	Source
Simulated	A4	V5	1,000,000	23	160-190	100	10-40	GemSIM (v4^#^)	[19,20]
	A5	V5	1,000,000	23	160-190	100	10-40	GemSIM (v5*^b^*)	[19,20]
	S4	V5	1,000,000	1	160	100	40	GemSIM (v4^#^)	[19,20]
	S5	V5	1,000,000	1	160	100	40	GemSIM (v5*^b^*)	[19,20]

Real	C1	V3	716,366	9	169-195	125	55-81	Illumina GAIIx	[21]
	C2	V3	1,350,602	9	169-195	125	55-81	Illumina GAIIx	[21]
	C3	V3	673,845	1	198	108	18	Illumina GAIIx	[10]

†hyper-variable regions in 16S rRNA; ‡the number of reference sequences; §excluding the primer (simulated).

#Illumina error model v4 (forward rate 0.99%, reverse 2.40%); *b*v5 (forward rate 0.28%, reverse 0.34%).

For performance comparison with the proposed CASPER method, we employ three widely used approaches to merging paired-end reads: COPE [11], FLASH [6], and PANDAseq [10]. All of the four tools compared take quality scores [9] as input, and the quality score information of each sequence used was prepared in the FASTQ format. The specification of the machine used is as follows: Ubuntu 12.04, 4*× *2.2 GHz Intel Xeon E5-4620 CPUs (8 cores/16 threads each), and 512 GB main memory.

### Performance evaluation and comparison

Table 3 lists the performance of CASPER and three other tools in terms of runtime (measured with 32 threads used) and other widely used metrics including the accuracy and *F*_1 _score [22]. We use the evaluation methodology proposed in [10], which examines the completeness of resolving mismatches in the overlap to call the success of a merge (refer to Additional file 1 for more details on the definition of evaluation metrics and additional experimental results).

**Table 3:** Performance statistics (A4, A5, S4, S5: simulated; C1, C2, PA: real)

Tool	dataset (# reads)	# merges	# correct merges	time (sec)	accuracy	*F*_1_	dataset (# reads)	# merges	# correct merges	time (sec)	accuracy	*F*_1_
**CASPER**		**999,936**	**967,842**	**30**	**0.968**	**0.984**		**713,782**	**667,421**	**23**	**0.932**	**0.965**
COPE	A4	262,661	241,630	183	0.242	0.389	C1	603,357	572,885	205	0.800	0.889
FLASH	(1,000,000)	989,960	732,040	20	0.732	0.845	(716,366)	688,730	601,561	22	0.840	0.913
PANDAseq		991,698	807,691	6	0.808	0.894		693,518	590,898	5	0.825	0.904

**CASPER**		**999,973**	**997,201**	**30**	**0.997**	**0.999**		**1,345,759**	**1,233,831**	**40**	**0.914**	**0.955**
COPE	A5	924,634	915,981	205	0.916	0.956	C2	1,105,743	1,046,420	319	0.775	0.873
FLASH	(1,000,000)	999,578	977,355	19	0.977	0.989	(1,350,602)	1,282,916	1,101,436	35	0.816	0.898
PANDAseq		999,101	978,527	6	0.979	0.989		1,298,903	1,080,593	9	0.800	0.889

**CASPER**		**1,000,000**	**960,986**	**29**	**0.961**	**0.980**		**671,877**	**658,631**	**19**	**0.977**	**0.989**
COPE	S4	262,107	230,595	181	0.231	0.375	PA	[COPE does not run on PA]				
FLASH	(1,000,000)	999,964	697,867	18	0.698	0.822	(673,845)	660,984	634,261	16	0.941	0.970
PANDAseq		999,976	785,919	5	0.786	0.880		660,593	635,663	4	0.943	0.971

**CASPER**		**1,000,000**	**997,303**	**28**	**0.997**	**0.999**						
COPE	S5	974,219	961,366	162	0.961	0.980						
FLASH	(1,000,000)	999,921	977,431	19	0.977	0.989						
PANDAseq		999,947	976,701	6	0.977	0.988						

Most notably, CASPER exhibits the highest level of accuracy and *F*_1 _score over all the datasets used. In particular, for A4/S4 (simulated with higher error rates) and C1/C2/PA (real data), the average accuracy of CASPER is higher than that of COPE, FLASH, and PANDAseq by 86%, 21%, and 15%, respectively. In terms of *F*_1 _scores, CASPER outperforms the other tools by 8-55% for A4/S4 and C1/C2/PA. For the simulated datasets with lower error rates (A5/S5), the performance of CASPER remains superior to the alternatives tested, although the performance gap is narrower due to the low error rates used to simulate A5 and S5. The evident robustness of CASPER would make it an invaluable tool for handling noisy sequencing data which are encountered frequently in practice. The performance of the tasks that utilize the paired-end merging results (such as genome assembly and mapping) will also benefit significantly from using CASPER, which can provide longer reads by merging forward and reverse reads robustly.

As for runtime, CASPER is not the fastest, as expected from the fact that CASPER relies on the time-consuming *k*-mer counting. PANDAseq and FLASH, which do not employ *k*-mer counts, normally take the least amount of runtime. Nonetheless, the runtime of CASPER is obviously reasonable in most cases (taking about thirty seconds), unlike COPE, another *k*-mer-based merger that takes noticeably more time than the *k*-mer-free tools. Clearly, there is a trade-off between the runtime and accuracy of the merging tools, but considering the quality of merges CASPER can provide, we believe that CASPER is the tool that best balances this trade-off.

### Experiments on the context definition and probability computation

**Figure 4:**
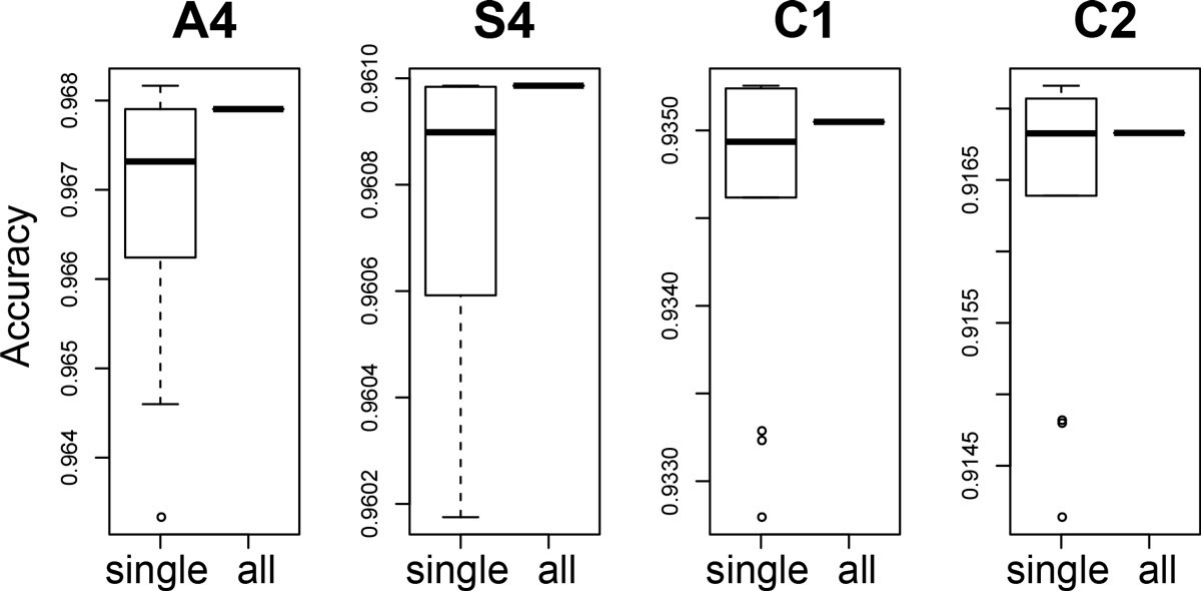
**Effects of making an integrative decision on the accuracy of merging**. CASPER makes a call based on the ensemble of up to *k *individual decisions. For comparison, we additionally implement and measure the performance a version that makes a decision based on only one of the *k *contexts. For each dataset, the box plot in the figure shows the distribution of the accuracy values this modified version reports for different *j *values (1 *≤ j ≤ k*).

In the context-based mismatch resolution, CASPER makes a call based on the ensemble of up to *k *individual decisions. For comparison, we additionally implement and measure the performance of a version that makes a decision based on only one of the *k *contexts, as shown in Figure 4. The distribution of the accuracy values this modified version reports for different *j *values (1 *≤ j ≤ k*) is represented by a box plot. We observe that the ensemble approach is effective for delivering more robust performance, although the observed performance gain is lower than the theoretical one (see 'Second pass: context-based correction' in Proposed Method), presumably due to the dependence between contexts.

As stated in 'Second pass: context-based correction' in Proposed Method, the context-based mismatch resolution of CASPER assumes that the bases in each of the forward and reverse reads have similar probabilities of being correct and thus does not consider quality scores when estimating probabilities using *k*-mer contexts. Alternatively, we can include quality scores in the probability estimation by multiplying the ratio of probabilities (*i.e*., $10^{-Q_{X}\left(i\right)/10}/10^{-Q_{Y}\left(i^{\prime }\right)/10}$) in Eq. (3) and Eq. (4). We implement both approaches and compare the results in Table 4 which indicates that there is negligible difference between these two approaches, and thus implying that the quality scores add little to the information provided by the *k*-mer contexts.

**Table 4:** Comparison of # correct merges with and without considering quality scores during the probability estimation using *k*-mer counts

dataset	# reads	#correct merges		increase by q-score (%)
		**(without)**	(with)	

A4	1,000,000	967,842	967,875	0.003410
A5	1,000,000	997,201	997,211	0.001003
S4	1,000,000	960,986	960,984	-0.000208
S5	1,000,000	997,303	997,303	0.000000
C1	716,366	667,421	667,165	-0.038357
C2	1,350,602	1,233,831	1,233,012	-0.066379
PA	673,845	658,631	658,648	0.002581

### Effects of algorithm parameters and multithreading on performance

#### Parameter k: the length of k-mer

In general, using larger *k *has the advantage of examining wider contexts, but on the other hand, demands more memory and runtime. In the literature, there exist many *k*-mer-based bioinformatics tools, and different values of *k *are recommended depending on tasks and applications. For instance, the default *k *for seed-and-extend aligners are 11 for BLAST [23] and 28 for MegaBLAST [24].

**Figure 5:**
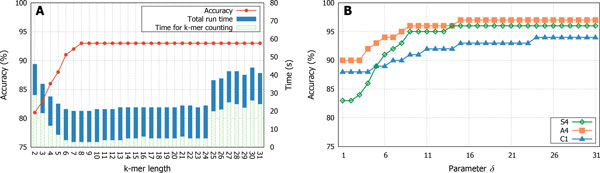
**Effects of parameters *k *and *δ *on performance**. (a) *k*, the size of *k*-mers defining contexts (dataset: C1). (b) *δ*, the threshold for starting context-based decisions (dataset: C1, S4, A4).

Figure 5(a) shows how the accuracy and runtime of CASPER varies as the *k*-mer size changes from *k *= 2 to *k *= 31 for dataset C1. To clearly show the overhead incurred by *k*-mer counting, Figure 5(a) also shows the time taken to count *k*-mers. In this figure, the accuracy of CASPER is seen to increase only up to a certain point as we increase *k*, plateauing out around *k *= 8. As *k *increases, the time to count *k*-mers starts decreasing at first but tends to increase in the long run, due to the IO bottleneck incurred by multithreading (see [14] for more details of this behavior). The only variation here is the value of *k*, and the total runtime of CASPER is directly proportional to the time taken to perform *k*-mer counting. The default value CASPER uses is *k *= 17, which is a widely used value in genome assembly [25]. Given the length of fragments in this C1 dataset, smaller values of *k *should work, as is observed in Figure 5(a). Users can change *k *depending on their analysis target.

#### Parameter δ: the threshold for starting context-based mismatch resolution

Recall from 'Second pass: context-based correction' in Proposed Method that CASPER makes context-based decisions on which of the forward and reverse reads is correct if the quality score difference falls below *δ*. Otherwise, CASPER utilizes the quality score difference for the decisions. Figure 5(b) presents the effects of *δ *on the accuracy of CASPER. For the datasets used (A4, S4, C1), we observe a similar trend: as *δ *increases, the accuracy improves and eventually becomes saturated. Note that the number of mismatches handled by the quality-score-based decision increases as we lower *δ*. Thus, the observation that the accuracy degrades as *δ *is lowered strongly implies that the quality-score-based scheme becomes ineffective as *δ *decreases. This observation justifies the switch to the context-based scheme when quality scores do not differ significantly between two mismatching bases. This is also compatible with our motivation for developing CASPER: simply relying on quality scores may incur mistakes in resolving mismatches, when the difference in quality scores is not significant.

#### Parameters γ and ω

**Figure 6:**
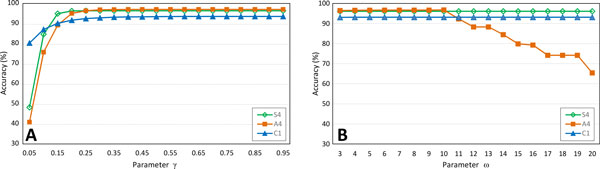
**Effects of parameters *γ *and *ω *on performance (dataset: C1, S4, A4)**. (a) CASPER abandons read merging if the mismatch ratio in the overlap is greater than *γ*. (b) *ω*, the minimum length (in bp) of the overlap region between forward and reverse reads.

CASPER abandons read merging if the mismatch ratio in the overlap region is greater than *γ*. Figure 6(a) shows how the accuracy of CASPER is affected by changing the *γ *threshold (data: C1, S4, A4). The values of the other parameters are set to their default. We can observe that the accuracy steeply increases as we change *γ *from 0 to approximately 0.2, but after that, the change in accuracy seems negligible.

Figure 6(b) shows how the accuracy changes as we vary the value of *ω *from 3 to 20 for the datasets C1, S4 and A4 (default values were used for the other parameters). In this plot, we do not see any significant change up to *ω *= 10. For larger values of *ω*, the accuracy gradually decreases for A4, whereas the accuracy does not change for the other datasets. This observation can be explained by noticing the size of overlaps in the datasets used. The overlap size is 40 for S4 and 50-81 for C1, as listed in Table 2. Consequently, there should be no difference in accuracy for these datasets by varying *ω *from 3 to 20. In contrast, the overlap size for A4 is between 10 and 40, and the accuracy of CASPER for A4 becomes affected if we increase *ω *over 10.

#### The number of computing threads used for parallelization

Considering that read pairs can be merged independently of each other, most of the current approaches to merging paired-end reads provide parallel implementations. The task of *k*-mer counting adds another opportunity for parallelization, and CASPER utilizes the parallelized version of Jellyfish [14] for constructing the *k*-mer-count table.

**Figure 7:**
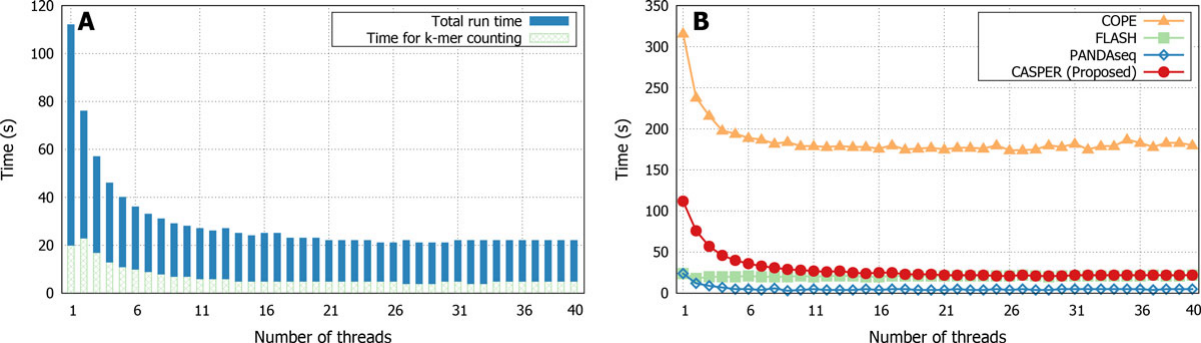
**Effects of the number of threads on runtime (dataset: C1)**. (a) the total runtime of CASPER and the time taken to *k*-mer counting only. (b) comparison of different tools.

Figure 7(a) shows how the runtime of CASPER decreases as more threads are used for parallel execution. Due to the part of the code that cannot be parallelized, the effect of multithreading diminishes as the number of threads increases past a certain point. The speedup of *k*-mer counting shows a similar trend, as can be observed in the plot.

Figure 7(b) shows a comparison of the four paired-end merging tools under comparison in terms of speedups by multithreading. For the cases where only a few threads are used, the *k*-mer-based methods (CASPER and COPE) are slower than FLASH and PANDAseq. As more threads are utilized, the runtime of CASPER steeply decreases and shows a similar run-time to the two non-*k*-mer approaches. In contrast, although the time demand of COPE is alleviated by multithreading to a certain extent, its runtime remains significantly higher than the others.

## Concluding remarks

Among the four merging tools tested, CASPER consistently shows the best performance in terms of accuracy and *F*_1 _score for the datasets used. We attribute the main reason for this improvement to the mismatching resolution policy employed by CASPER: when the quality scores differ significantly, it trusts the scores and uses them to decide the correct bases; otherwise, it switches to the context-based decision scheme based on *k*-mer counts around the mismatching bases. CASPER is most clearly differentiated from other approaches by this policy.

One might suggest incorporating quality scores to some extent even when CASPER makes a context-based decision, similarly to an existing approach [11]. For instance, we could adjust the *k*-mer counts by considering the quality scores of the bases in a *k*-mer. According to our tests (*e.g*., see Table 4), however, it is difficult to see any significant effect of this hybrid approach on the accuracy of merging once the quality score difference becomes lower than a threshold value.

The most notable findings from our experiments can thus be summarized as follows: when the difference between the quality scores of a pair of mismatching bases falls below a threshold, the quality scores often fail to deliver decisive information on the identity of the correct base. Instead, using the *k*-mer contexts around the mismatching position is believed to be more informative.

We envision various opportunities for improving CASPER: (1) Currently, the overlap finding step and the mismatch resolution step run separately. Given that a suboptimal arrangement of the overlap may become optimal after the mismatches therein are corrected, a future revision of CASPER may include simultaneous or iterative optimization of these two steps. (2) The context-based decision of CASPER presently relies on the idea of voting and may further be improved by adopting other types of ensemble learning techniques. In the revision process, care should be taken not to incur excessive computational overhead for learning. (3) CASPER presently considers only substitution type sequencing errors. Although substitutions are the most common error type in Illumina sequencing, indels (especially in homopolymers) are still problematic intervals particularly at read ends. By revising the context-based mismatch-resolution scheme, we may augment CASPER so that it can recognize and address indel-type sequencing errors as well as substitutions. (4) For the current form of dependence on *k*-mer counts, CASPER is particularly well suited for high-coverage amplicon sequencing. According to our experiments (summarized in Additional file 1), however, the degree of sequencing depth required to obtain a high accuracy with CASPER tends to be moderate, suggesting its wide applicability of CASPER. For whole-genome shotgun sequencing, the context around mismatches in the overlap between forward and reverse reads may be defined differently to optimize the effectiveness of CASPER. It would be intriguing to analyze the effect of running CASPER on a variety of downstream tasks such as sequence assembly/mapping and metagenomic diversity estimation.

## Authors' contributions

SK implemented the method, carried out the experiments, and analyzed the results. BL participated in the experiments and analyzed the results. SY conceived the research, analyzed the results, and wrote the manuscript. All authors read and approved the final manuscript.

## Competing interests

The authors declare that they have no competing interests.

## 

**input **: *X, Y, Q_X _, Q_Y _*// *X*: forward read, *Y *: reverse read, *Q_X _,Q_Y _*: q-scores

**param **: *ω, γ, δ, k *// *ω*: min overlap, *γ*: give-up threshold, *δ*: difference threshold, *k*: *k*-mer size

**output**: *Z *// virtually elongated read by merging forward & reverse reads

// step 3: find the best overlapping region between forward & reverse reads

**1 **lowestMismatchRatio ← ∞, bestOverlapStartIndex ← 0

**2 for ***i ← *1 **to ***n − ω *+ 1 **do**

**3**    numMismatches ← 0

**4**    **for***j ← i ***to*** n ***do**

**5**      **if**X(j) ≠ Y (j − i + 1) **then **numMismatches ← numMismatches + 1

**6**    $\frac{numMismatches}{n-i+1}$*< lowestMismatchRatio ***then**

**7**    *lowestMismatchRatio←*$\frac{numMismatches}{n-i+1}$

**8**    bestOverlapStartIndex ← i

**9 if ***lowestMismatchRatio > γ ***then **return // give up (too many mismatches)

// step 4 (first pass): qualty-score-based mismatch resolution

**10 for **i ← bestOverlapStartIndex **to **n **do**

**11**    *i′ *= *i − bestOverlapStartIndex *+ 1 // for convenience in indexing *Y*

**12**    **if***X*(*i*) = *Y *(*i′*) **then **continue // skip matching bases

**13**    **else if ***Q_X_*(*i*) *− Q_Y_*(*i′*) *> δ ***then ***Y *(*i′*) = *X*(*i*) // q in *X *is significantly better

**14**    **else if ***Q_Y_*(*i′*) *− Q_X_*(*i*) *> δ ***then ***X*(*i*) = *Y*(*i′*) // q in *Y *is significantly better

// step 4 (second pass): context-based mismatch resolution

**15 for **i ← bestOverlapStartIndex **to **n **do**

**16 ***i′ *= *i − bestOverlapStartIndex *+ 1 // for convenience in indexing *Y*

**17 if ***X*(*i*) = *Y *(*i′*) **then **continue // skip matching bases

**18 ***ForwardVotes ← *0, *ReverseVotes ← *0 // otherwise, examine the *k*-mer context

**19 for ***j ← *1 **to ***k ***do**

**20**    **if ***i − k *+ *j <*1 **then **continue // index exceeds read boundary (left); continue to next *j*

**21**    **if ***i *+ *j − *1 *> n ***then **break // index exceeds read boundary (right); go to line 27

**22**    **if ***j >*1 **and ***X*(*i *+ *j − *1) *≠ Y *(*i′ *+ *j − *1) **then **break // context scan stops; go to line 27

**23**    *Context_X _← T_k_*[*X*(*i − k *+ *j *: *i *+ *j − *1)] // *k*-mer counts in forward reads

**24**    *Context_Y _← T_k_*[*Y *(*i′ − k *+ *j *: *i′ *+ *j − *1)] // *k*-mer counts in reverse reads

**25**    **if **Context_X _> Context_Y _**then **ForwardVotes ← ForwardVotes + 1

**26**    **else if **Context_X _< Context_Y _**then **ReverseVotes ← ReverseVotes + 1

**27 if ***ForwardVotes > ReverseVotes ***then ***Y*(*i′*) *← X*(*i*) // use the base in *X*

**28 else ***X*(*i*) *← Y *(*i′*) // use the base in *Y*

// step 5: merge forward & reverse reads

**29 for ***i ← *1 **to ***bestOverlapStartIndex − *1 **do ***Z*(*i*) *← X*(*i*) // copy first part from *X*

**30 for ***i ← *1 **to ***n ***do ***Z*(*i *+ *bestOverlapStartIndex − *1) *← Y *(*i*) // copy second part from *Y*

**31 **return *Z*

**Algorithm 1: **Steps 3-5 of the proposed CASPER algorithm

## Supplementary Material

Additional file 1 (PDF): description of performance evaluation methods and additional results. Click here for file

## Declarations

Funding for open access charge was supported by Seoul National University.

This article has been published as part of BMC Bioinformatics Volume 15 Supplement 9, 2014: Proceedings of the Fourth Annual RECOMB Satellite Workshop on Massively Parallel Sequencing (RECOMB-Seq 2014). The full contents of the supplement are available online at http://www.biomedcentral.com/bmcbioinformatics/supplements/15/S9.
