# Supplementary material for: CASPER: context-aware scheme for paired-end reads from high-throughput amplicon sequencing
Source: BMC Bioinformatics. 2014 Sep 10;15(Suppl 9):S10. doi: 10.1186/1471-2105-15-S9-S10 (PMC4168710; doi:10.1186/1471-2105-15-S9-S10)
Supplement: Additional file 1 — (PDF): description of performance evaluation methods and additional results. [file 1471-2105-15-S9-S10-S1.pdf]

CASPER: context-aware scheme for paired-end read from high-throughput amplicon sequencing

Sunyoung Kwon[1], Byunghan Lee[2], and Sungroh Yoon[1,2*]

[1] *Interdisciplinary Program in Bioinformatics, Seoul National University, Seoul 151-747, Korea*
[2] *Electrical and Computer Engineering, Seoul National University, Seoul 151-744, Korea*

[Supplementary information]

S1 Performance evaluation methods

To assess the performance of the four tools compared in the paper, we use the evaluation methodology proposed in [1], which calls the success of a merge according to the completeness of mismatch resolution in the overlap region (see Fig. S1). Specifically, we formulate the determination whether a merge is successful or not as a binary classification problem. We define a true positive (TP) as a merge that correctly resolves all the mismatching bases in the overlap region with respect to the reference sequence. A false positive (FP) is defined as a merge with incorrect mismatching resolution in the overlap region. A false negative (FN) is a merge that escapes detection by CASPER. A true negative (TN) is undefined in this context.

In terms of the definitions of TP, FP and FN, accuracy and F_1 scores (the two widely used performance metrics) are defined as follows [2]:

$$\text{accuracy} = \frac{\#\text{TP}}{\#\text{TP} + \#\text{FP} + \#\text{FN}} \tag{1}$$

$$F_1 \text{ score} = 2 \cdot \frac{\text{precision} \cdot \text{recall}}{\text{precision} + \text{recall}} \tag{2}$$

where

$$\text{precision} = \frac{\#\text{TP}}{\#\text{TP} + \#\text{FP}} \tag{3}$$

$$\text{recall} = \frac{\#\text{TP}}{\#\text{TP} + \#\text{FN}}. \tag{4}$$

S1.1 An alternative evaluation method

Note that there is another way of defining true/false positives/negatives in the literature, as shown in Figure S1. Given that the novelty of CASPER lies in resolving mismatching bases in overlapping regions (rather than finding overlaps per se), the main text uses the 'Label definition I' scheme shown in Figure S1 for performance comparison. It is also possible to use the 'Label definition II' depicted in Figure S1 for evaluating CASPER and the other three methods. In this labeling scheme, true negatives are defined as correct predictions of the reads that do not truly overlap, and the definition of accuracy becomes

$$\text{accuracy} = \frac{\#\text{TP} + \#\text{TN}}{\#\text{TP} + \#\text{TN} + \#\text{FP} + \#\text{FN}} \tag{5}$$

*To whom correspondence should be addressed: sryoon@snu.ac.kr

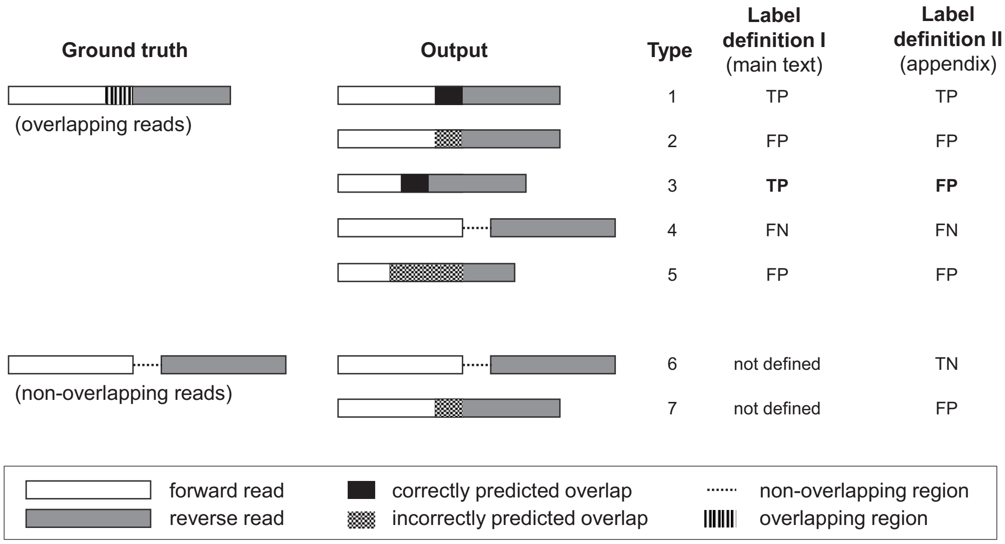

Figure S1: Definitions of labels included in performance metrics. 'Label definition I' was proposed in [1] and used in the main text, whereas 'Label definition II' was proposed in [3] and used in Appendix. For output types 1–5, the forward and reverse reads overlap; for output types 6–7, there is no overlap between the reads. For types 1 and 2, the length of the fragment is correctly predicted, but the predicted overlap is correct only for type 1. For type 3, the bases in the overlap region are correctly predicted, but the location of overlap is incorrectly predicted. For types 4 and 5, the overlap is either not detected or incorrectly predicted. Types 6 and 7 are not defined in Label definition I.

while the definition of the F_1 score remains unchanged.

Table S1 lists the performance statistics evaluated using the 'Label definition II' scheme for the same datasets used in the main text. This result shows that CASPER consistently produces the best accuracy and F_1 scores for both of the labeling schemes depicted in Figure S1.

S2 Additional experimental results

S2.1 Performance comparison for datasets with non-overlapping reads

We carried out additional experiments to show the performance of CASPER for datasets with true negatives (*i.e.*, non-overlapping reads). To this end, we first created a new dataset called N4 using nearly the same method used to create the four simulated datasets presented in the main text (A4/A5/S4/S5). That is, we used the GemSIM (v4) model to simulate 1,000,000 reads (100 bp each) from twenty three reference seqeunces originating from the V5 region of bacterial 16S rRNAs. However, the fragment length was set to 200–250 in N4 so that forward and reverse reads do not overlap. We then mixed N4 with A4 and C2 in turn, generating two mixed datasets in which a number of forward and reverse reads do not overlap. The results from applying CASPER and the other three tools to these mixture datasets are presented in Table S2.

According to this result, CASPER maintains its superiority to the compared alternatives in terms of accuracy and F_1 score even for the datasets with many non-overlapping reads. The ability of CASPER to discover overlaps is similar to that of the alternatives (*i.e.*, similar amounts of TNs except PANDAseq in Table S2), but CASPER outperforms the other methods in terms of correcting

Table S1: Performance statistics using alternative definitions of TP/TN/FP/FN labels

tool	dataset (# reads)	# merges	# correct merges	time (sec)	accuracy	F_1	dataset (# reads)	# merges	# correct merges	time (sec)	accuracy	F_1
**CASPER**		**999,936**	**965,440**	**30**	**0.965**	**0.982**		**713,782**	**627,923**	**23**	**0.877**	**0.934**
COPE	A4	262,661	240,965	183	0.241	0.388	C1	603,357	546,159	205	0.762	0.865
FLASH	(1,000,000)	989,960	730,227	20	0.730	0.844	(716,366)	688,730	570,992	22	0.797	0.887
PANDAseq		991,698	805,551	6	0.806	0.892		693,518	562,391	5	0.785	0.880
**CASPER**		**999,973**	**994,748**	**30**	**0.995**	**0.997**		**1,345,759**	**1,160,385**	**40**	**0.859**	**0.924**
COPE	A5	924,634	913,687	205	0.914	0.955	C2	1,105,743	997,128	319	0.738	0.849
FLASH	(1,000,000)	999,578	974,989	19	0.975	0.987	(1,350,602)	1,282,916	1,045,379	35	0.774	0.873
PANDAseq		999,101	976,093	6	0.976	0.988		1,298,903	1,028,110	9	0.761	0.864
**CASPER**		**1,000,000**	**959,788**	**29**	**0.960**	**0.979**		**671,877**	**632,522**	**19**	**0.939**	**0.968**
COPE	S4	262,107	230,304	181	0.230	0.374	PA	[COPE does not run on PA]				
FLASH	(1,000,000)	999,964	696,999	18	0.697	0.821	(673,845)	660,984	610,204	16	0.906	0.950
PANDAseq		999,976	784,958	5	0.785	0.880		660,593	611,262	4	0.907	0.951
**CASPER**		**1,000,000**	**995,627**	**28**	**0.996**	**0.998**						
COPE	S5	974,219	959,790	162	0.960	0.979						
FLASH	(1,000,000)	999,921	975,821	19	0.976	0.988						
PANDAseq		999,947	975,093	6	0.975	0.987						

Parameters: $k = 17, \omega = 10, \gamma = 0.5, \delta = 19$; machine: Ubuntu 12.04, Intel Xeon E5-4620×4, 512-GB memory; these statistics were computed in terms of the 'Label definition II' in Figure S1.

Table S2: Performance statistics for datasets with non-overlapping reads

tool	dataset (# reads)	# merges	TP	FP	FN	TN	accuracy	F_1
**CASPER**	A4	992,857	958,004	34,853	7,160	999,983	**0.979**	**0.979**
COPE	(1,000,000)	261,783	240,365	21,418	738,223	999,994	0.620	0.388
FLASH	+ N4	989,978	730,227	259,751	10,040	999,982	0.865	0.844
PANDAseq	(1,000,000)	1,463,590	805,551	658,039	8,302	528,108	0.667	0.707
**CASPER**	C2	1,289,902	1,153,053	136,849	60,717	999,983	**0.916**	**0.921**
COPE	(1,350,602)	1,105,749	997,128	108,621	244,859	999,994	0.850	0.849
FLASH	+ N4	1,282,934	1,045,379	237,555	67,686	999,982	0.870	0.873
PANDAseq	(1,000,000)	1,770,795	1,028,111	742,684	51,699	528,108	0.662	0.721

Parameters: $k = 17, \omega = 10, \gamma = 0.27, \delta = 19$; machine: Ubuntu 12.04, Intel Xeon E5-4620×4, 512-GB memory; these performance statistics were calculated using the 'Label definition II' in Figure S1.

mismatches in the overlap (*i.e.*, better performance in terms of TPs, FPs, and FNs), yielding the best accuracy and F_1 scores overall.

S2.2 Effects of sequencing depth on the accuracy of CASPER

For the current form of dependence on k-mer counts, CASPER is suited primarily to high-coverage amplicon sequencing due to the need for counting k-mer information. To determine the level of sequencing coverage required to achieve a high performance of CASPER, we measured the accuracy of CASPER as the sequencing depth is varied from 1 to 500 for the simulated data created from the A4 dataset. Figure S2 shows the result. As expected, the performance improves as we increase the sequencing depth. However, after a certain point (in this case, a sequencing depth of approximately five), the performance improvement is no longer noticeable. This experimental result suggests that CASPER is applicable not only for high-coverage amplicon sequencing data but also for moderate-coverage data.

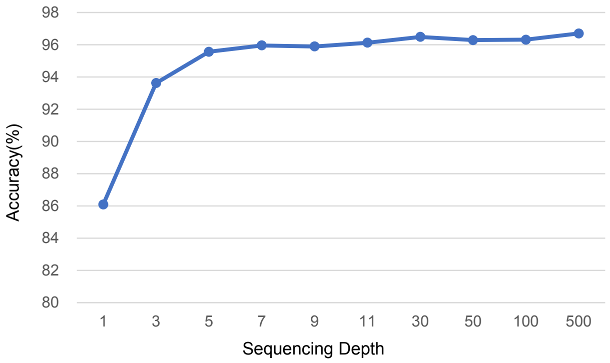

Figure S2: Effect of sequencing depth on accuracy. The accuracy of CASPER improves as the sequencing depth increases and becomes saturated when the depth is 5 or higher.

References

[1] A. P. Masella, A. K. Bartram, J. M. Truszkowski, D. G. Brown, and J. D. Neufeld, "PANDAseq: paired-end assembler for Illumina sequences," *BMC Bioinformatics*, vol. 13, no. 1, p. 31, 2012.

[2] I. H. Witten and E. Frank, *Data Mining: Practical machine learning tools and techniques*. Burlington, Massachusetts: Morgan Kaufmann, 2005.

[3] B. Liu, J. Yuan, S.-M. Yiu, Z. Li, Y. Xie, Y. Chen, Y. Shi, H. Zhang, Y. Li, T.-W. Lam, *et al.*, "COPE: an accurate k-mer-based pair-end reads connection tool to facilitate genome assembly," *Bioinformatics*, vol. 28, no. 22, pp. 2870–2874, 2012.
